# Appropriateness of psychotropic drug prescriptions in the elderly: structuring tools based on data extracted from the hospital information system to understand physician practices

**DOI:** 10.1186/s12913-019-4064-7

**Published:** 2019-04-30

**Authors:** Aurélie Petit-Monéger, Vianney Jouhet, Frantz Thiessard, Driss Berdaï, Pernelle Noize, Véronique Gilleron, Guillaume Caridade, Louis-Rachid Salmi, Florence Saillour-Glénisson

**Affiliations:** 1 CHU de Bordeaux, Pôle de santé publique, Service d’Information Médicale, F-33000 Bordeaux, France; 2 Univ. Bordeaux, ISPED, Centre INSERM U1219-Bordeaux Population Health, F-33000 Bordeaux, France; 3 INSERM, ISPED, Centre INSERM U1219-Bordeaux Population Health, F-33000 Bordeaux, France; 4 CHU de Bordeaux, Pôle de santé publique, Service de Pharmacologie Médicale, F-33000 Bordeaux, France

**Keywords:** Automated indicators, Drug prescription surveillance system, Psychotropic drugs, Physicians’ practices, Quality of care

## Abstract

**Background:**

The appropriateness of psychotropic prescriptions in the elderly is a major quality-of-care challenge at hospital. Quality indicators have been developed to prevent inappropriate psychotropic prescriptions. We aimed to select and automatically calculate such indicators, from the Bordeaux University Hospital information system, and to analyze the appropriateness of psychotropic prescription practices, in an observational study.

**Methods:**

Experts selected indicators of the appropriateness of psychotropic prescriptions in hospitalized elderly patients, according to guidelines from the French High Authority for Health. The indicators were reformulated to focus on psychotropic administrations. The automated calculation of indicators was analyzed by comparing their measure to data collected from a clinical audit. In elderly patients hospitalized between 2014 and 2015, we then analyzed the evolution of the appropriateness of psychotropic prescription practices during hospital stay, using methods of visualization, and described practices by considering patients’ characteristics.

**Results:**

Two indicators were automated to detect overuse and misuse of psychotropic drugs. Indicators identified frequent inappropriate drug administrations, but practices tended to become more appropriate after quality-of-care improvement actions. In the majority of patients (85%), there was no inappropriate administration of psychotropic drugs during hospital stay; for the remaining 15% with at least one inappropriate administration, physicians tended to limit overuse or misuse during hospital stay. Inappropriate administrations were more frequent in patients suffering from psychiatric disorders, dependence and associated complications or morbidities.

**Conclusions:**

The automated indicators are structuring tools for the development of a drug prescription monitoring system. Inappropriate psychotropic administrations were limited by physicians during hospital stay; some inappropriate prescriptions might be explained by clinical characteristics of patients.

**Electronic supplementary material:**

The online version of this article (10.1186/s12913-019-4064-7) contains supplementary material, which is available to authorized users.

## Background

Appropriateness of care is defined as the adequacy of any care to patient needs, in accordance with practice guidelines [1]. In the elderly, appropriateness of drug use holds a special place due to chronic diseases and degenerative disorders that can lead to a higher drug consumption [2]. Inappropriate drug prescriptions, which refer to the use of drug when the risk of care outweighs the clinical benefit [3], are associated with negative outcomes [4, 5], including adverse drug reactions [6], hospitalizations [7] and deaths [8]. As the prevalence of drug adverse events is high in hospitalized elderly patients [9], improving the appropriateness of drug prescriptions could significantly reduce such events at hospital.

Explicit criteria have been published to improve the appropriateness of drug use in the elderly [3, 10–13]. International studies reported that 30 to 60% of drug prescriptions could be inappropriate in hospitalized elderly patients, depending on the type of criteria considered and characteristics of study populations [4, 14, 15]. Thus, improvement of the appropriateness of drug prescriptions in the elderly is a major challenge to improve quality and safety of care at hospital.

As psychotropic drugs are among the drugs most frequently involved in adverse events [9, 16], specific guidelines and related Clinical Practice Indicators (CPIs) have been developed by the French High Authority for Health to help prevent inappropriate psychotropic prescriptions [17–21]. These CPIs constitute a panel of quality indicators focusing on professional practice related to psychotropic prescriptions in the elderly. Indicators are operational tools for identifying at-risk situations and implementing quality-of-care improvement actions, considering barriers to guideline implementation by physicians [22].

Clinical audits are often used to assess the conformity to guidelines of observed clinical practices. Nevertheless, they do not allow a follow-up if indicators are not implemented to assure continuous monitoring and regular feedback, as recommended to improve practices [23]. At Bordeaux University Hospital, we have implemented a strategy that focuses on the construction of tools such as automated CPIs to identify wards needing an improvement of the drug prescription appropriateness. As an application of this strategy, the first aim of the study was to select and calculate CPIs automated from the hospital information system, focusing on potential inappropriate psychotropic prescriptions. The second aim of the study was to analyze the evolution of the appropriateness of psychotropic prescription practices during hospital stays of elderly patients and to describe practices by considering patients’ characteristics, using data extracted from the hospital information system.

## Methods

This observational study, while not covered by French regulation governing biomedical research and routine care during its conduct [24], fully complied with ethical principles laid down in the Declaration of Helsinki as well as applicable provisions for handling and protecting individual personal data. The Publication Group of the Ethics Committee of the Bordeaux University Hospital approved the publication of this research work (GP – CE 2018/06).

### Study population and structure of the study

The study population included patients aged over 75 who were hospitalized at the Bordeaux University Hospital between 2014 and 2015 and who were administered at least one psychotropic drug during their hospital stay.

This exploratory study was carried out in four steps: 1) selection, by experts, of CPIs to be implemented to describe the appropriateness of psychotropic prescriptions in hospitalized elderly; 2) calculation and automatization of the selected indicators from the hospital information system and analysis of their agreement with results of a clinical audit; 3) analysis of the practices of psychotropic prescription by considering their evolution during patients’ hospital stays, from data extracted from the hospital information system; 4) analysis of psychotropic prescription practices depending on patients’ characteristics, from data extracted from the hospital information system.

### Step 1: selection of quality indicators by experts

A multi-disciplinary group of experts, including geriatricians, epidemiologists, pharmacologists, pharmacists and specialists of the hospital information system practicing at the Bordeaux University Hospital, was established locally by considering their experience in that fields. The main role of these experts was to select CPIs that would be implemented to describe the appropriateness of psychotropic prescriptions in hospitalized elderly patients, according to guidelines from the French High Authority for Health. The process of CPI selection, which focused on indicators designed to alert to the existence of potential inappropriate psychotropic prescriptions in the elderly, was performed from the list of CPIs developed by the French High Authority for Health [17–21] with respect to the level of scientific evidence and clinical expertise from national learned societies in geriatrics [25]. Based on a consensus method including face-to-face meetings and a qualitative group synthesis [26], the experts prioritized indicators depending on their potential utility (defined as their ability to detect inappropriate psychotropic prescriptions and identify improvement actions), their operational implementation in terms of frequency or severity (defined as their ability to detect frequent or severe inappropriate psychotropic prescriptions allowing practice improvement) and their operational feasibility for implementation using the hospital information system (defined as the availability of data within the hospital information system for measuring the indicator). The selected indicators were then adapted by experts to focus on the assessment of psychotropic administrations (psychotropic prescribed and administered) rather than only on psychotropic prescriptions (psychotropic prescribed without systematically being administered), while taking into account their face validity (defined as their suitability (or not) to meet the objective of the measure). This approach aimed at identifying potential inappropriate prescriptions that had led to potential inappropriate drug administrations.

### Step 2: calculation of automated quality indicators and analysis of their agreement with a clinical audit

The indicators were automatically calculated, from the Bordeaux University Hospital information system, for patients aged over 75 who were hospitalized from 1st of January 2014 to 31th of December 2014. Their calculation was based on data focusing on patients’ socio-demographic characteristics (especially the age of patients) and data on patients’ prescriptions and administrations of psychotropic drugs during hospital stay (including long-half-life benzodiazepines). They were calculated at a hospital level and in geriatric medicine wards previously concerned by a clinical audit performed at Bordeaux University Hospital to improve the appropriateness of drug prescriptions in the elderly [27]. This clinical audit was performed in a long-term care unit to analyze the appropriateness of targeted drugs commonly prescribed in the elderly and based on the above-mentioned CPIs developed by the French High Authority for Health [28]. The automated indicators were then analyzed by comparing their measure to data collected from 60 electronic patient records within the hospital information system, randomly sampled for the clinical audit; the latter was performed from March 2014 (first assessment of drug prescriptions’ practices) to July 2014 (re-assessment of drug prescriptions’ practices after the implementation of active quality-of-care improvement actions in May 2014). The improvement actions consisted of: (i) reinforcing the dissemination of prescription guidelines among geriatricians and; (ii) organizing meetings between geriatricians and pharmacists, to optimize prescriptions and reinforce data collection in the electronic patient record by considering patients’ clinical specificities in the elderly (for example the collection of creatinine clearance or patients’ weight). This analysis was also used as a descriptive tool to check whether trends in changes of practice reported by the automated indicators agreed with trends reported by results of the audit before and after implementation of these actions.

### Step 3: analysis of the evolution of psychotropic prescription practices during hospital stays

Psychotropic prescription practices during hospital stays of elderly patients were analyzed, from the Bordeaux University Hospital information system, by using methods of visualization. We considered the flow of inappropriate administrations, taking patients’ hospital stay as the unit of analysis; this allowed describing practices across different hospital wards. We considered patients aged over 75 who had received at least one psychotropic administration from 1st of January 2014 to 31th of December 2014. We then distinguished four different patients’ statuses: (i) patients without any inappropriate administration (less than three psychotropic drugs and no long half-life benzodiazepine); (ii) patients with at least one administration of three psychotropic drugs without any long-half-life benzodiazepine; (iii) patients with at least one administration of a long-half-life benzodiazepine but less than three psychotropic drugs; (iv) patients with at least one administration of three psychotropic drugs among which there was at least one long-half-life benzodiazepine. A Sankey diagram was used to visualize the sequential switches between different patients’ statuses [29, 30]; this diagram reports the evolution of status, represented by colored rectangles, connected by a curve representing the change from one status to another, whose thickness is proportional to the number of changes of status. Only patients for whom there was at least one change of status during hospital stay are represented.

### Step 4: analysis of psychotropic prescription practices depending on patients’ characteristics

Based on a case-control study design, we analyzed psychotropic prescription practices depending on patients’ characteristics, which were extracted from medico-administrative data of the Bordeaux University Hospital information system. Data focused on patients’ socio-demographic and hospital stay characteristics as well as diseases, signs and symptoms coded with the International Statistical Classification of Diseases and Related Health Problems 10th Revision. Among patients aged 75 and over who were hospitalized in medical or surgical wards at the Bordeaux University Hospital from 1st of July 2014 to 31th of July 2015, cases were defined as patients who were administered at least two psychotropic drugs and one long half-life benzodiazepine during hospital stay. Among cases, we compared patients for whom the inappropriate co-administration was received more than once (case group 1) to patients for whom the inappropriate co-administration was received only once (case group 2); severity during hospital stay was described on a numerical scale from 1 (no associated complication or morbidity) to 4 (high level of associated complication or morbidity), as defined from data of the information system. Cases were compared to controls aged 75 and over who were not administered such an inappropriate co-administration during hospital stay; this control group was further divided into four control subgroups: (i) patients who were administered two psychotropic drugs without any long half-life benzodiazepine (control group 1); (ii) patients who were administered one psychotropic drug without any long half-life benzodiazepine (control group 2); (iii) patients who were administered at least one long half-life benzodiazepine without any other psychotropic drug (control group 3) and; (iv) patients who were not administered any long half-life benzodiazepine nor any other psychotropic drug during their hospital stay (control group 4). Patients who had been hospitalized in psychiatry or rehabilitation medicine wards were excluded from the analysis, as the data needed were not available in the information system.

### Statistical analyses

Qualitative variables were described with frequencies and percentages; they were compared with chi-squared tests. Quantitative variables were described with means and standard deviations for a normal distribution, with medians and percentiles otherwise; they were compared with student tests or non-parametric tests depending on sample size (< 30 or not) and depending on the variable distribution (normal distribution or not). A level of 5% was considered for statistical significance.

## Results

### Step 1: selection of quality indicators by experts

All solicited experts agreed to participate to the study’s first step. Two CPIs, developed by the French High Authority for Health [18, 19], were selected by the experts: (i) the ratio between the number of elderly patients with a prescription of at least three psychotropic drugs (including long-half-life benzodiazepines) and the total number of elderly patients considered and; (ii) the ratio between the number of elderly patients with a prescription of at least one long-half-life benzodiazepine and the total number of elderly patients considered.

After an adaptation of these selected indicators by experts to focus on drug administrations, indicator 1, which aimed at detecting overuse of psychotropic drugs, was defined as the ratio between the number of patient-days in elderly aged over 75 with an administration of at least three psychotropic drugs on the same day during hospital stay (including long-half-life benzodiazepines) and the total number of patient-days in elderly aged over 75 hospitalized between 2014 and 2015. Indicator 2, which aimed at detecting misuse of psychotropic drugs, was defined as the ratio between the number of patient-days in elderly aged over 75 with an administration of at least one long-half-life benzodiazepine during hospital stay and the total number of patient-days in elderly aged over 75 hospitalized between 2014 and 2015. These indicators had to be extractable from the hospital information system using only drug administration information, without requiring any other clinical data.

### Step 2: calculation of automated quality indicators and analysis of their agreement with a clinical audit

**Fig. 1:**
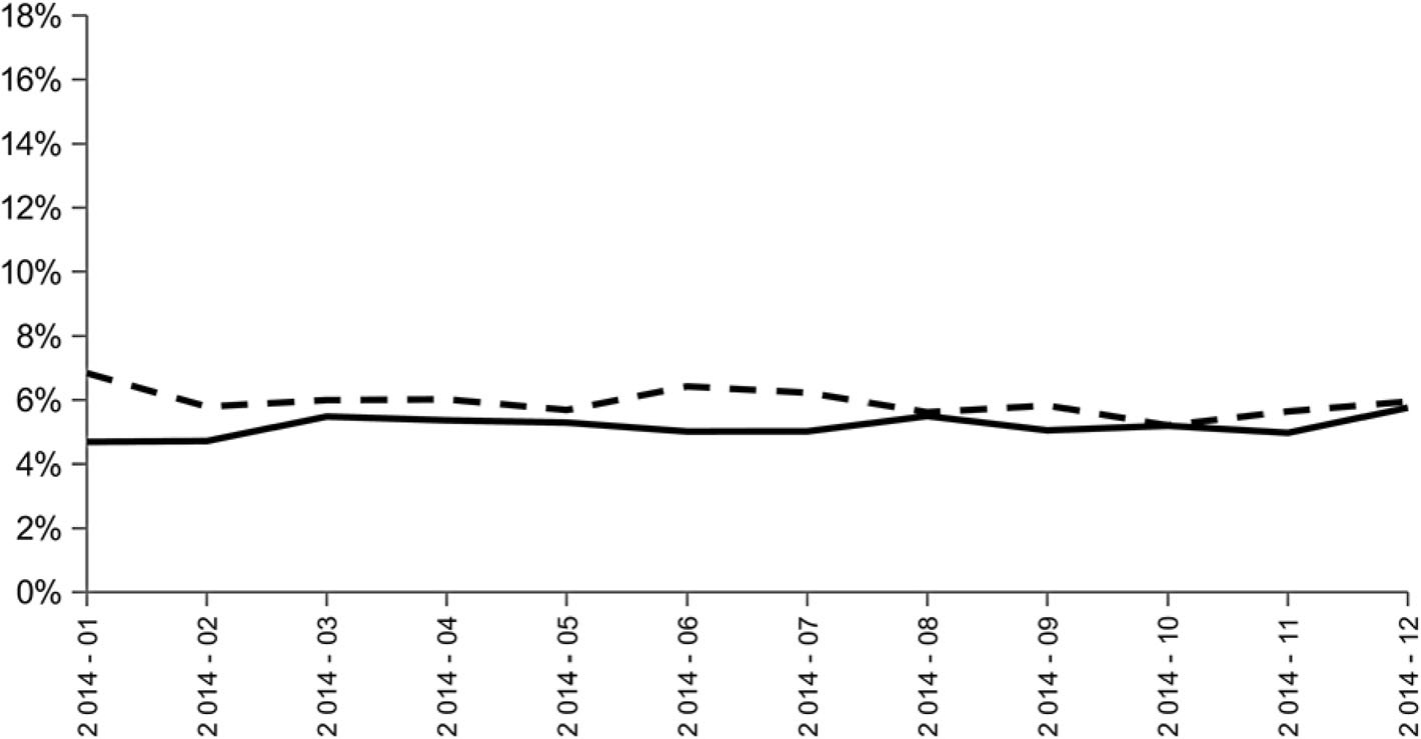
Evolution of the ratio between the number of patient-days in elderly aged over 75 with an administration of at least three psychotropic drugs on the same day during hospital stay (including long-half-life benzodiazepines) and the total number of patient-days in elderly aged over 75 hospitalized between 2014 and 2015 (indicator 1: black line) and the ratio between the number of patient-days in elderly aged over 75 with an administration of at least one long-half-life benzodiazepine during hospital stay and the total number of patient-days in elderly aged over 75 hospitalized between 2014 and 2015 (indicator 2: black dotted line) at Bordeaux University Hospital; Bordeaux, from January 2014 to December 2014

At the hospital level, the two indicators were calculated from data on 16,234 patients; this corresponded to 283,039 patient-days, 511,526 drug prescriptions and 3,692,595 drug administrations. Results of indicators 1 and 2 did not report any trend toward improvement of the appropriateness of psychotropic drugs and long half-life benzodiazepine administrations from January to December 2014 (Fig. 1).

**Fig. 2:**
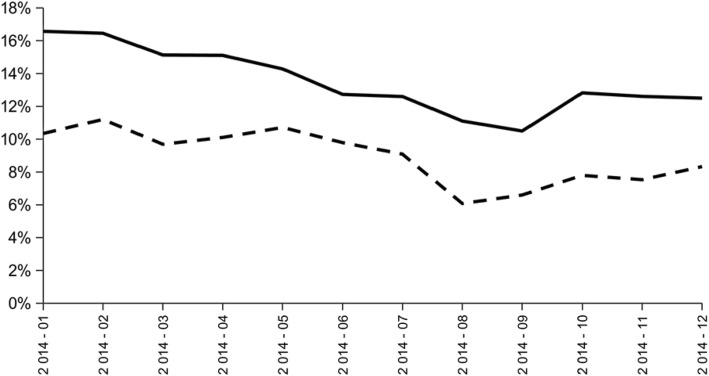
Evolution of the ratio between the number of patient-days in elderly aged over 75 with an administration of at least three psychotropic drugs on the same day during hospital stay (including long-half-life benzodiazepines) and the total number of patient-days in elderly aged over 75 hospitalized between 2014 and 2015 (indicator 1: black line) and the ratio between the number of patient-days in elderly aged over 75 with an administration of at least one long-half-life benzodiazepine during hospital stay and the total number of patient-days in elderly aged over 75 hospitalized between 2014 and 2015 (indicator 2: black dotted line) at Bordeaux University Hospital’s long-term geriatric medicine wards; Bordeaux, from January 2014 to December 2014

At the geriatric ward level, the two indicators were calculated from data on 131 patients; this corresponded to 34,796 patient-days, 8979 drug prescriptions and 478,314 drug administrations. There were frequent inappropriate administrations of psychotropic drugs and long half-life benzodiazepine (Fig. 2). A trend toward improvement of the appropriateness of psychotropic administrations was observed, with a decrease from 17 to 13% of inappropriate administrations from January to December 2014. There was a less significant trend toward improvement of the appropriateness of long half-life benzodiazepine administrations from January to December 2014. Frequencies of inappropriate administrations were at their lowest level in August and September 2014, following the implementation of active quality-of-care improvement actions but tended to increase again as early as October 2014. These observations were judged in agreement with the results of the clinical audit by physicians from the geriatric wards who clearly identified a decline in active quality-of-care improvement actions at the end of 2014 due to physician turnover.

### Step 3: analysis of the evolution of psychotropic prescription practices during hospital stays

From 1st of January 2014 to 31th of December 2014, 303,800 administrations were reported in 17,394 patients. During hospital stay, 85% of patients did not have any inappropriate administration (less than 3 psychotropic drugs, and no long half-life benzodiazepine). The majority of the remaining 15% had two changes of status during their hospital stay: first, from a status of appropriate administration to a status of potential inappropriate administration, and then a return to a status of appropriate administration (Sankey diagram in Additional file 1).

We observed a strong evolution toward return to appropriate administrations during hospital stay, in patients with potential inappropriate administrations; only a limited proportion of patients had still a potentially inappropriate administration at the end of hospital stay. There was an improvement during hospital stay for all statuses: (i) 65% of patients were not administered any inappropriate psychotropic administration at the beginning of hospital stay, compared to 86% at the end of hospital stay; (ii) 27% of patients were administered at least three psychotropic drugs without any administration of long half-life benzodiazepine at the beginning of hospital stay, compared to 10% at the end of hospital stay; (iii) 6% of patients were administered at least one long half-life benzodiazepine but less than three psychotropic drugs at the beginning of hospital stay, compared to 3% at the end of hospital stay; (iv) 2% of patients were administered at least three psychotropic drugs including at least one long half-life benzodiazepine at the beginning of hospital stay, compared to 1% at the end of hospital stay.

### Step 4: analysis of psychotropic prescription practices depending on patients’ characteristics

**Fig. 3:**
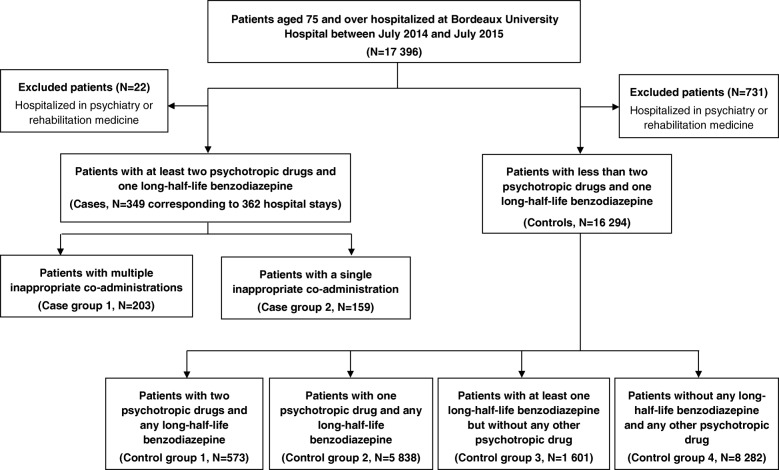
Flow chart of patients’ selection process at Bordeaux University Hospital; Bordeaux, from July 2014 to July 2015

The case group (*N* = 349, corresponding to 362 hospital stays) included 203 group-1 cases and 159 group-2 cases. The control group (*N* = 16,294) included 573 group-1 controls, 5838 group-2 controls, 1601 group-3 controls and 8282 group-4 controls (Fig. 3).

The median age was 83. In cases, patients with multiple inappropriate co-administrations had significantly longer median length of stay (case group 1: 24 days) than patients with a single inappropriate co-administration (case group 2: 16 days) (*p* < 0.05) (Table 1). Group-1 cases were more frequently managed for mental disorders (26%) than group-2 cases (15%) (*p* < 0.05); they also had more frequently mood disorders (35% versus 23%) and were administered more frequently the inappropriate co-administration at hospital discharge (6% versus 1%) (*p* < 0.05). Similarly, there was a high proportion of hospital stays with a high level of severity (> 2) in both group (case group 1: 68%; case group 2: 57%). The first day at hospital, 30 patients (14%) received multiple inappropriate co-administrations and 10 patients (7%) received a single inappropriate co-administration. These inappropriate practices tended to decrease during hospital stay; at the last day of hospital stay, 12 patients (6%) received multiple inappropriate co-administrations and only 2 patients (1%) received a single inappropriate co-administration.

**Table 1:** Characteristics of hospital stays of patients aged 75 and over who were administered at least two psychotropic drugs and one long half-life benzodiazepine more than once (case group 1, *N* = 203) compared to hospital stays of patients aged 75 and over who were administered at least two psychotropic drugs and one long half-life benzodiazepine only once (case group 2, *N* = 159) during stay; Bordeaux, from July 2014 to July 2015

Characteristics	Case group 1 (*N*=203)		Case group 2 (*N*=159)	
	N	%	N	%
Gender of the patient				
Male	86	42.4	53	33.3
Female	117	57.6	106	66.7
Stay with high level of severity				
Yes	137	67.5	91	57.2
No	66	32.5	68	42.8
Management for mental disorders^a^	53	26.1	23	14.5
Psychiatric disorders^b^				
Dementia	81	39.9	57	35.8
Cognitive disorders	70	34.5	55	34.6
Mood disorders^a^	71	35.0	36	22.6
Anxiety disorders	62	30.5	45	28.3
Psychoses and personality disorders	39	19.2	25	15.7
Alcohol addiction	16	7.9	6	3.8

^a^Statistically significant difference (level of significance = 5%)

^b^Codes of the International Statistical Classification of Diseases and Related Health Problems 10th Revision (ICD-10): Dementia (F00, F01, F02, F03); Cognitive disorders (R41, F06.7); Mood disorders (F32, F33, F38, F39); Anxiety disorders (F40, F41, F42, F43); Psychoses (F20, F21, F22, F23, F24, F25, F28, F29) and personality disorders (F60, F61, F62, F63, F64, F65, F66, F68); Alcohol addiction (F10)

There were more women in cases (60%) than in controls (54%) (*p* < 0.05), and the case group also had more psychiatric disorders such as dementia (36% versus 11%), mood disorders (25% versus 7%) or anxiety disorders (27% versus *7*%) (*p* < 0.05). Patients in the case group were also more dependent (39% versus 16%) with longer median lengths of stay (20 days versus 6 days) and higher level of severity for hospital stay (70% versus 35% with a level of severity > 2) (*p* < 0.05) (Table 2). Prescriptions were most frequently inappropriate when patients presented psychiatric disorders and associated complications or morbidities. Comparisons with the four control groups are presented in Table 3.

**Table 2:** Characteristics of patients aged 75 and over who were administered at least two psychotropic drugs and one long half-life benzodiazepine (cases, *N* = 349) compared to patients aged 75 and over who were not administered such a prescription (controls, *N* = 16,294) during their hospital stay; Bordeaux, from July 2014 to July 2015

Characteristics	Cases (*N*=349)		Controls (*N*=16294)	
	N	%	N	%
Gender^a^				
Male	139	40.0	7450	45.7
Female	210	60.0	8844	54.3
Stay with high level of severity^a^				
Yes	245	70.2	5698	35.0
No	104	29.8	10596	65.0
Primary diagnosis during stay^b^				
Cognitive symptoms^a^	42	12.0	316	1.9
Vascular dementia^a^	20	5.7	53	0.3
Mood symptoms^a^	18	5.2	31	0.2
Psychiatric disorders^b^				
Dementia^a^	127	36.4	1750	10.7
Mood disorders^a^	93	26.6	1155	7.1
Anxiety disorders^a^	94	26.9	1177	7.2
Alcohol addiction^a^	26	7.4	340	2.1
Dependence^a^	137	39.3	2580	15.8
Social status				
Living alone at home^a^	21	6.0	492	3.0
Requiring nursing home entry^a^	18	5.2	295	1.8

^a^Statistically significant difference (level of significance = 5%)

^b^Codes of the International Statistical Classification of Diseases and Related Health Problems 10th Revision (ICD-10): Cognitive symptoms (R41); Vascular Dementia (F01); Mood symptoms (R45); Dementia (F00, F01, F02, F03); Mood disorders (F32, F33, F38, F39); Anxiety disorders (F40, F41, F42, F43); Alcohol addiction (F10)

**Table 3:** Characteristics of patients aged 75 and over who were administered at least two psychotropic drugs and one long-half-life benzodiazepine (cases, *N* = 349) compared to four control groups of patients aged 75 and over who were not administered such a prescription during their hospital stay; Bordeaux, from July 2014 to July 2015

Characteristics	Cases (*N*=349)		Control group 1^a^ (*N*=573)		Control group 2 ^b^ (*N*=5838)		Control group 3 ^c^ (*N*=1601)		Control group 4^d^ (*N*=8282)	
	N	%	N	%	N	%	N	%	N	%
Gender										
Male	139	40.0	193	33.7	2514	43.1	763	47.7	3980	48.1
Female	210	60.0	380	66.3	3324	56.9	838	52.3	4302	51.9
Stay with high level of severity										
Yes	245	70.2	383	66.8	2794	47.9	751	46.9	1770	21.4
No	104	29.8	190	33.2	3044	52.1	850	53.1	6512	78.6
Primary diagnosis during stay^e^										
Cognitive symptoms	42	12.0	41	7.2	131	2.2	40	2.5	104	1.3
Vascular dementia	20	5.7	10	1.7	20	0.3	6	0.4	17	0.2
Mood symptoms	18	5.2	4	0.7	14	0.2	8	0.5	5	0.1
Psychiatric disorders^e^										
Dementia	127	36.4	167	29.1	795	13.6	193	12.1	595	7.2
Mood disorders	93	26.6	179	31.2	627	10.7	132	8.2	217	2.6
Anxiety disorders	94	26.9	168	29.3	569	9.7	197	12.3	243	2.9
Alcohol addiction	26	7.4	21	3.7	145	2.5	43	2.7	131	1.6
Dependence	137	39.3	213	37.2	1153	19.7	370	23.1	844	10.2
Social status										
Living alone at home	21	6.0	32	5.6	241	4.1	46	2.9	173	2.1
Requiring nursing home entry	18	5.2	27	4.7	161	2.8	43	2.7	64	0.8

^a^Control group 1: patients who were administered two psychotropic drugs without any long half-life benzodiazepine

^b^Control group 2: patients who were administered one psychotropic drug without any long half-life benzodiazepine

^c^ Control group 3: patients who were administered at least one long half-life benzodiazepine without any psychotropic drugs

^d^ Control group 4: patients who were not administered any psychotropic drugs nor any long half-life benzodiazepine

^e^Codes of the International Statistical Classification of Diseases and Related Health Problems 10th Revision (ICD-10): Cognitive symptoms (R41); Vascular Dementia (F01); Mood symptoms (R45); Dementia (F00, F01, F02, F03); Mood disorders (F32, F33, F38, F39); Anxiety disorders (F40, F41, F42, F43); Alcohol addiction (F10)

## Discussion

### Main results

This study illustrates an innovative dynamic approach using data extracted from the hospital information system to highlight drug prescription practices in elderly hospitalized patients. Two selected indicators, whose calculation was automated from the hospital information system to detect overuse and misuse of psychotropic drugs, identified frequent inappropriate drug administrations and were useful to follow up the appropriateness of these drugs; practices tended to be improved by reinforcing the implementation of prescription guidelines and clinical pharmacy actions. Furthermore, the visualization methods and analysis of patients’ characteristics highlighted that physicians tended to limit overuse or misuse of psychotropic drugs during hospital stays and that some inappropriate administrations might be explained by clinical specificities of the patients.

### Comparison with the literature

The results observed for the prevalence of inappropriate prescriptions in hospitalized elderly patients, at the beginning of their hospital stay, are consistent with Gallagher et al study, who applied Beers’ and STOPP criteria [4] in elderly patients admitted to six European hospitals and observed a prevalence of potential inappropriate long half-life benzodiazepine prescriptions between 1 and 7% across hospitals. Our results are also consistent with Gobert et al who reported that 67% of patients in nursing homes in Quebec, where access to nursing homes is strongly regulated and patients are admitted when home care is no longer possible, did not use any psychotropic drug and that 21% of patients used at least two different families of psychotropic drugs [31].

Gallagher et al showed a significant association between the prescription of a STOPP-listed potential inappropriate medicine and female gender, cognitive impairment and increasing number of medications [4]. These results are also consistent with our results reporting that patients who had received at least two psychotropic drugs and one long half-life benzodiazepine were more frequently women, with cognitive disorders or dementia, and suffering from associated complications or morbidities.

### Strengths

The study developed methods for automatically calculating indicators from the hospital information system; their measures did not need any ad hoc pro-active data collection. The development of such automated methods is fundamental to produce regular feedback to health professionals and improve practices [23]. As the use of indicators of the appropriateness of drug prescriptions in daily hospital practice is lacking, these tools should reinforce the role of health professionals at the heart of the quality and safety improvement process [32].

This study is the first, to our knowledge, based on the development of an innovative dynamic approach to highlight the evolution of drug prescription practices throughout hospital stay. It is innovative in that it combines the automated calculation of indicators with methods of visualization of potential inappropriate administrations, using a Sankey diagram, to highlight the sequential switches between different drug administration statuses. This original approach was completed by the description of hospital stay characteristics in elderly patients. Thus, the present study provides important knowledge about potential mechanisms of overuse or misuse management by physicians for psychotropic drug prescriptions during elderly patient hospital stays.

Furthermore, there are very few studies in France related to practices of psychotropic drug prescription and administration. Such studies often used an administrative database in the general population [33], which allows the inclusion of large study samples but only report drugs reimbursed by health insurances and not drugs actually administered. This reinforces the strength of an approach based on administrative data extracted from the hospital information system.

### Limits

This exploratory study was first implemented in a single university hospital center to ensure feasibility, based on experience of professionals and data extracted from the hospital information system. Thus, we chose preferentially to develop such an innovative approach locally, at the Bordeaux University Hospital, by soliciting a multi-disciplinary group of experts with a high level of experience in the evaluation of drug prescriptions’ practices in the elderly.

One could argue that a limit of the study is the process of indicators’ selection by experts. This selection was first based on a consensus method with a qualitative group synthesis, but we paid great attention to assess the potential utility, operational implementation and face validity of the indicators during organized face-to-face meetings in a group of experienced experts. This selection was also based on CPIs developed by the French High Authority for Health and their ad hoc adaptation to focus on drug administration. Furthermore, these indicators aimed at detecting overuse and misuse of psychotropic drugs, both in their initial or adapted definition, in accordance with the scientific evidence as reported by the French High Authority for Health [18, 19]. Even if the selected French guidelines do not synthesize all available knowledge, this should not preclude our approach to be applicable to other guidelines and related CPIs. In this sense, the generalization of the study findings seems to be relatively preserved, especially for the overall approach that we propose to highlight the appropriateness and evolution of drug prescription practices during hospital stay.

This observational study was developed as an operational system to identify potential need for quality-of-care improvement actions. After selecting and calculating automated indicators, our approach was based on the analysis of prescription practices during hospital stay and on the analysis of prescription practices depending on patients’ characteristics. For this last analysis, we performed univariate statistical analyses as an operational tool to highlight prescription practices but further analyses using multivariate logistic regression models will be needed to identify factors associated with inappropriate prescriptions and potential confounding factors. Following an exploratory approach, analyses were performed without sample size calculations.

### Interpretation of the results and applicability

Results of the indicators automated at the geriatric ward level confirmed the potential impact of active quality-of-care improvement actions to reinforce the appropriateness of drug prescriptions in the elderly. Even if such measures need to be maintained over time to ensure sustainable changes in physicians’ practices, the improvement of drug prescriptions’ appropriateness, especially psychotropic drugs, could significantly reduce negative outcomes such as drug adverse events in hospitalized elderly patients [9].

The analysis of psychotropic prescription practices, using data extracted from the hospital information system, highlighted that physicians tended to limit overuse or misuse of psychotropic during hospital stays, with a high proportion of patients with appropriate administrations at hospital discharge. Some inappropriate administrations might be explained by clinical specificities of the patients who had more frequently psychiatric disorders, dependence and associated complications or morbidities. We hypothesize that some practices might be explained by the need for physicians to manage acute decompensation of mental disorders for which patients were hospitalized or managed for at the beginning of hospitalization; the fact that practices were more appropriate at the end of hospitalization might reflect that disorders would then be under control. Nevertheless, as these inappropriate administrations are potentially due to a non-compliance of prescriptions with guidelines, the automated indicators are structuring tools for the follow up of the appropriateness of psychotropic drugs and the implementation of quality-of-care improvement actions. As the use of indicators and feedback increases as a method to improve practices, this approach could be used as a model for the analysis of the appropriateness of any other prescription practices from the hospital information system, in any healthcare institution in which data are available and interested in improving quality of care.

## Conclusion

These automated quality indicators are structuring tools for the development of a drug prescription surveillance system in hospitalized elderly patients. Combined with methods of visualization and description of patients’ characteristics during hospital stay, they allow making dynamic analysis of trends for prescription practices over time and highlighting clinical specificities that might explain some inappropriate prescriptions. As such, this approach is a good warning system to detect the need for quality-of-care improvement actions and strengthen the ability of healthcare institutions answering to current institutional requirements in quality of care, in France or elsewhere. For appropriateness of care, this approach could be extended to other domains, especially for measuring and analyzing the appropriateness of biological or radiological prescriptions as well as appropriateness of hospitalizations.

## Additional file

Additional file 1: Sequential changes of patient’s status according to the administration of psychotropic drugs at Bordeaux University Hospital; Bordeaux, from January 2014 to December 2014. (TIFF 6979 kb)

## Abbreviation

CPIs: Clinical Practice Indicators
